# Nonlinear Physical Growth of Children from Infancy to Middle Adolescence in Low- and Middle-Income Countries

**DOI:** 10.34172/jrhs.2021.69

**Published:** 2021-11-14

**Authors:** Senahara Korsa Wake, Temesgen Zewotir, Essey Kebede Muluneh

**Affiliations:** ^1^Department of Statistics, College of Sciences, Bahir Dar University, Bahir Dar, Ethiopia; ^2^School of Mathematics, Statistics and Computer Sciences, University of KwaZulu-Natal, Durban, South Africa; ^3^School of Public Health, Bahir Dar University, Bahir Dar, Ethiopia

**Keywords:** Growth curves, Longitudinal data, Mixed-effect model, Physical growth

## Abstract

**Background:** The growth curve has a significant role in understanding the growth trajectories over time and examining the mathematical relationship between the outcome variable and time.

**Study design:** A longitudinal prospective cohort study.

**Methods:** This study aimed to identify a nonlinear growth curve that best represents the growth trajectories in children’s physical growth from ages 1 to 15 years. The data were obtained from the Young Lives study conducted in Ethiopia, India, Peru, and Vietnam. Nonlinear growth curves were studied through the families of three-parameter nonlinear mixed-effects models.

**Results:** The study examined the performances of different three-parameter nonlinear growth curves for the growth trajectory analysis, and the Logistic curve was chosen for the trajectory analysis. Gender and country differences had significant effects on the child’s growth. Females reached asymptotic height earlier and shorter than males. The mean height values at the end of the growth stage for children in Ethiopia, India, Peru, and Vietnam were 171.78, 170.37, 171.30, 174.31cm, respectively. Children in Ethiopia approached adult height earlier than those in India but later than children in Peru. However, no significant growth change was observed between children in Ethiopia and Vietnam. This indicates that children in Ethiopia and Vietnam have no significant differences regarding approaching adult height.

**Conclusion:** The study concludes that the Logistic curve was found to be the best growth curve to describe the growth trajectories. Children in all four countries exhibited different growth parameters.

## Introduction

The motivation to study the growth of children is to adequately describe the basic biological process of physical growth and monitor nutritional status, cognitive development, and health outcomes. Growth curves have a significant role in understanding and modeling the growth of children. Repeated measures observed on the same outcomes over time are the starting points for growth curves^1,2^. To analyze such a design, a longitudinal study offers a more realistic view of growth patterns at individual and group levels. In longitudinal studies, individuals are observed repeatedly on the same outcome over time^3,4^. Observations repeatedly measured on the same outcome at multiple occasions tend to be inter-correlated. This correlation must be taken into account in the analysis since failing to do so could result in inaccurate and inefficient inferences. A key requirement for longitudinal data analysis is to accurately estimate the random effects of the model so that the underlying mean and individual functions can be efficiently modeled^3,5^.

The physical growth of children from infancy to adulthood follows nonlinear growth patterns^6,7^. Nonlinear mixed-effect models are a generalized form of linear mixed-effects models in which the within-individual model relating the outcome variable to time is nonlinear in the parameters^8^. When the goal is to characterize nonlinear changes over time, nonlinear mixed-effects models are commonly employed in longitudinal studies. Variations and correlations among repeated measurements can be partitioned into between-individual and within-individual variations. As a consequence, a nonlinear model provides better predictions outside the range of observed data, and its parameters usually have natural physical interpretations. Moreover, nonlinear models use a small number of parameters, compared to the linear models^9,10^.

Low- and middle-income countries are characterized by huge socioeconomic inequality. Socioeconomic inequalities in physical growth are frequently observed with shorter height in lower socioeconomic groups^11,12^. The attainment of knowledge on the child*’*s growth is essential for effective management and feeding practices. This study aimed to examine the nonlinear height growth of children from infancy to 15 years of age and find a reasonable nonlinear growth curve that properly and parsimoniously described their trajectories.

## Methods

###  Data source

The data were obtained from the Young Lives study that examines the changing nature of childhood poverty and health in Ethiopia, India, Peru, and Vietnam. The study followed two prospective cohort groups, namely the older and younger cohorts. An older cohort of 1,000 children born before the millennium development goals and a younger cohort of 2,000 children born just after the millennium development goals were recruited from each country. Details regarding sampling and participant recruitment were discussed in previously published studies^13,14^. For this study, the outcome of interest is a periodic child’s physical height measured in centimeters from ages 1 to 15 years. A child’s height was collected by rounds every three/four years over a time of 15 years from 2002 to 2016. The younger children were tracked from 1 to 15 years, at ages 1, 5, 8, 12, and 15 years, and older children were tracked from 8 to 22 years, at ages 8, 12, 15, 19, and 22 years^15,16^.

###  Participants 

 The inclusion criteria of the study participants were children in a younger cohort with complete height measurements. Accordingly, this study included children who had height measured five times between the ages of 1 and 15 from each country. On the other hand, the children who had less than five times height measurements were excluded from the study. A total of 33,005 observations were obtained from 6,601 children from all four countries, each of which was measured five times. The obtained data were analyzed using SAS statistical software (version 9.4). Formal ethical approval for this study was obtained from the Young Lives study which was reviewed and approved by the Ethics Committee of Oxford University.

###  Statistical analysis

 A nonlinear mixed-effects model that follows a specified nonlinear function was used to analyze the change of outcomes over time. The model has two stages, and in the first stage, the j^th^ observation on the i^th^ subject is modeled as:


(1) $$y_{ij}=f(\phi_{ij},x_{ij})+\varepsilon_{ij},\;i=1,2,...,m\;\&j=1,2,...,n_{i}$$

where y_ij_ is the j^th^ repeated observation on the i^th^ individual, f(.) signifies a known nonlinear function of a subject-specific parameter vector ϕ_ij_, and the covariate vector x_ij_, ε_ij_ is represents a normally distributed random error, m indicates the number of individuals, and n_i_ is the number of observations on the i^th^ individual. In the second stage, the subject-specific parameter vector is modeled as:


(2) $$\phi_{ij}=A_{ij}\beta +B_{ij}b_{i\;},\;b_{i}\sim N(O,D)$$

where β is a vector of fixed effects, b_i_ signifies random effects vector associated with the i^th^ individual, A_ij_ and B_ij_ are design matrices for the fixed and random effects, respectively, and D represents a variance-covariance matrix. The random effects and error terms are identically and normally distributed with mean zero and variance-covariance [i.e., b_i_~N(0,D), ε_ij_~N(0,R_i_)]; moreover, b and ε are assumed to be independent. The first- and second-stage models of (1) and (2) can also be expressed in the matrix form as:


$$\left.\begin{array}{l} y_{i}=f_{i}\left(\phi_{i},X_{i}\right)+\varepsilon_{i}\\ \phi_{i}=A_{i}\beta +B_{i}b_{i} \end{array}\right\},\;i=1,2,...,m$$

where


$$\begin{array}{l} y_{i}={\left[y_{i1}\;...y_{in_{i}}\right]}^{\prime }\\ \varepsilon_{i}={\left[\varepsilon_{i1}\;...\varepsilon_{in_{i}}\right]}^{\prime }\\ f_{i}\left(\phi_{i},X_{i}\right)={\left[f\left(\phi_{i1},x_{i1}\right)\;...\;f\left(\phi_{in_{i}},x_{in_{i}}\right)\right]}^{\prime }\\ X_{i}={\left[{x^{\prime }}_{i1}:...:{x^{\prime }}_{in_{i}}\right]}^{\prime }\\ A_{i}={\left[{A^{\prime }}_{i1}:...:{A^{\prime }}_{in_{i}}\right]}^{\prime }\\ B_{i}={\left[{B^{\prime }}_{i1}:...:{B^{\prime }}_{in_{i}}\right]}^{\prime } \end{array}$$

The intra-individual and inter-individual variations are separately quantified by the variance components of random effects (D) and error term (R_i_). The covariance matrix D measures the inter-individual (between-individual) variation that is not explained by covariates, whereas the covariance matrix R_i_ measures the within-individual variation.

The physical growth of children has an evident asymptote. Polynomial and other parsimonious linear models are unsuitable when the mean response asymptotes to an upper or lower bound. For such situations, a nonlinear growth curve is required to fit well the data. The most popular growth curves with an asymptote are the three-parameter growth curves^17^. For this study, the Logistic, Von Bertalanffy, Brody, and Gompertz growth curves are considered to analyze the growth trajectories. The mathematical expressions for growth curves with random effects for all parameters are as follows:

 Logistic curve:


(3) $$y_{ij}=\frac{\beta_{1}+b_{1i}}{1+\left(\beta_{2}+b_{2i}\right)\exp\left(-\left(\beta_{3}+b_{3i}\right)t_{ij}\right)}+\varepsilon_{ij}$$

Brody curve:


(4) $$y_{ij}=\left(\beta_{1}+b_{1i}\right)\left(1-\left(\beta_{2}+b_{2i}\right)\exp\left(-\left(\beta_{3}+b_{3i}\right)t_{ij}\right)\right)+\varepsilon_{ij}$$

Gompertz curve:


(5) $$y_{ij}=\left(\beta_{1}+b_{1i}\right)\exp\left(-\left(\beta_{2}+b_{2i}\right)\exp\left(-\left(\beta_{3}+b_{3i}\right)t_{ij}\right)\right)+\varepsilon_{ij}$$

Von Bertalanffy curve:


(6) $$y_{ij}=\left(\beta_{1}+b_{1i}\right){\left(1-\left(\beta_{2}+b_{2i}\right)\exp\left(-\left(\beta_{3}+b_{3i}\right)t_{ij}\right)\right)}^{3}+\varepsilon_{ij}$$

In all models presented, y_ij_ stands for the physical height of children at age t, β_1_ is the asymptotic height, β_2_ signifies the predicted value at t=0, and β_3_ represents a constant related to the postnatal rate of growth that means the rate at which child growth approaches asymptotically; furthermore, b_1i_, b_2i_, and b_3i_ are the random effects associated with the fixed effects in the population, and they are assumed to be independent and identically distributed with mean zero and variance-covariance matrix D. In addition, ε_ij_ are the random errors assumed to be independent and identically distributed with mean zero and variance-covariance matrix R.

###  Parameter estimation 

The maximum likelihood and restricted maximum likelihood techniques are methods suggested for estimating parameters in nonlinear mixed models^18^. The estimation procedures were generated using the SAS PROC NLMIXED procedure. The best growth curve model was determined based on the Akaike information criterion (AIC) and Schwarz-Bayesian information criterion (BIC)^19^. It is worth mentioning that the lower AIC leads to a better model.

## Results

**Figure 1:**
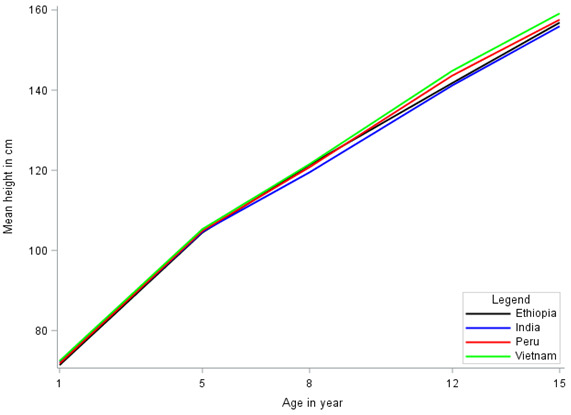
Mean profile plot of height growth by countries

**Figure 2:**
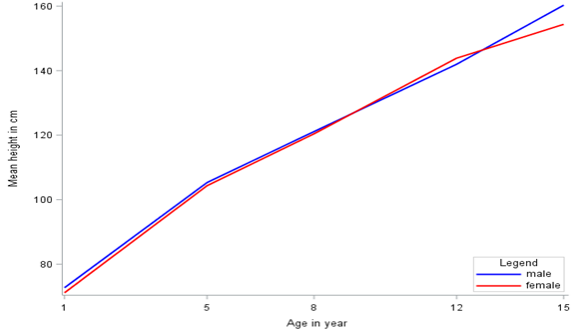
Mean profile plot of height growth by gender

The descriptive statistics of the physical height of children by gender and country are given in Table 1. The mean height is increased with age; however, the increment is not constant. The mean height of males is higher than that of females at ages 1, 5, 8, and 15; moreover, females had a higher mean height at age of 12 years in all countries. The patterns of mean height growth by gender and country are displayed in Figures 1 and 2, respectively. These figures confirmed that the growth trajectories follow nonlinear trends. The mean plots presented in Figures 1 and 2 are important in understanding the functional relationship between the mean height and time (a child’s age). From these plots, it can be observed that the functional relationship is not linear. A nonlinear growth curve is therefore a reasonable curve to analyze the growth trajectories.

**Table 1:** Descriptive statistics for five repeated measures of height for males and females in Ethiopia, India, Peru, and Vietnam

**Continuous** **variables**	**Ethiopia**				**India**				**Peru**				**Vietnam**			
	**Male**		**Female**		**Male**		**Female**		**Male**		**Female**		**Male**		**Female**	
	**Mean**	**SD**	**Mean**	**SD**	**Mean**	**SD**	**Mean**	**SD**	**Mean**	**SD**	**Mean**	**SD**	**Mean**	**SD**	**Mean**	**SD**
Age (yr)																
1	72.03	5.19	70.67	5.40	72.86	4.75	71.36	4.74	72.57	4.34	71.36	4.74	73.31	4.17	71.49	4.02
5	104.96	4.86	103.94	5.27	104.86	4.46	104.41	4.64	105.53	5.76	104.26	5.82	105.91	5.12	104.71	4.55
8	121.75	5.55	120.92	5.92	119.77	5.29	119.17	5.69	121.19	5.49	120.37	5.59	121.87	5.88	121.20	5.56
12	141.02	5.91	142.61	7.13	140.17	6.55	142.36	6.80	142.71	7.29	144.61	6.65	144.04	8.16	145.69	7.24
15	157.44	7.99	156.22	5.98	158.94	7.56	152.52	5.35	161.65	6.48	153.47	4.96	162.98	6.47	155.16	5.31
**Categorical** **variables**	**n**	**%**	**n**	**%**	**n**	**%**	**n**	**%**	**n**	**%**	**n**	**%**	**n**	**%**	**n**	**%**
Number of participants	818	51.7	764	48.3	877	53.2	771	76.8	819	50.3	808	49.7	887	50.9	857	49.1

**Figure 3:**
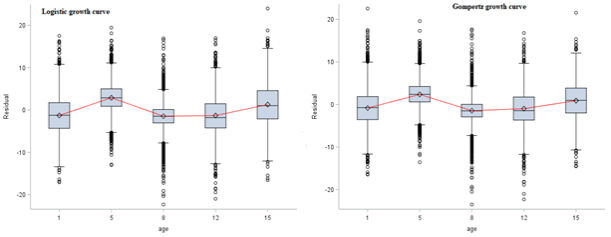
Trends of residual distributions for Logistic (the left panel) and Gompertz (the right panel) growth curves

Each growth curve yields different values of growth parameters. The Brody and Gompertz curves had the highest average asymptotic height of 237.25 and 196.00, respectively, while the Von Bertalanffy and Logistic curves had the lowest average asymptotic height of 119.58 and 180.27, respectively. Even though the Gompertz curve exhibited the lower AIC and BIC, the mean asymptotic height provided by this model is less practical in terms of biological expectations and does not appropriately determine the value associated with adult height. An alternative approach in determining the best model is to plot the residual distributions of different models and compare patterns of distribution^22^. The residual distribution of the Gompertz curve follows a similar pattern to that of the Logistic curves (Figure 3). This suggests that both Logistic and Gompertz curves can achieve good growth trajectories. Aside from using AIC and BIC for model selection, it is also vital to balance a theoretical framework with biologically relevant parameters^23^. Therefore, the Logistic curve approach was favored to analyze the current longitudinal data since its growth parameters have physical meaning and are biologically interpretable.

Different growth curves were applied to describe physical growth and age relationships in the context of nonlinear mixed-effects models, and their performances were compared. It is crucial to assess which growth parameters in the model should include a random component and whether the variance-covariance matrix of the random effects can be constructed in a simpler form with fewer parameters. The presence of these random effects was determined by comparing the AIC and BIC for non-nested models and using the likelihood ratio tests for the nested models^20,21^.

It was found that the inclusion of b1i and b3i as random effects in the model improved the fitting performance of the growth curves. Table 2 presents the estimated parameters and goodness of fit results. This table shows that the Gompertz curve revealed the smallest values of AIC=200681 and BIC=200729. However, the Von Bertalanffy model does not seem appropriate for the description of the growth data at hand as it had the largest information criteria (AIC=310986 and BIC=311033), compared to other growth curves.

**Table 2:** Fitted values of nonlinear mixed-effects growth curves for physical height

** Parameter/fit-statistics**	**Growth curve**			
	**Logistic**	**Gompertz**	**Brody**	**Von Bertalanffy**
Asymptote	180.27	196.00	237.25	119.58
Scale parameter	1.7182	1.1011	0.7312	-210.47
Rate of growth	0.1609	0.1059	0.0513	273.08
AIC	202542	200681	206965	310986
BIC	202589	200729	207013	311033

Once a proper growth curve is chosen, the evaluation of the nonlinear growth trajectory with the effect of covariates is the next task. The parameter estimates of the final best fit model with covariate effects added to all the growth curves are presented in Table 3. The fitted marginal Logistic curve is described by the following function:

 Height=A1+Bexp-Ctij

 where

 A=β1+β11gender+β1kcountry

 B=β2+β21gender+β2kcountry

 C=β3+β31gender+β3kcountry

 k is a dummy variable that represents the levels of the country (Ethiopia, India, Peru, and Vietnam).

**Table 3:** Estimates of the best fit mixed-effects growth curve with the effects of gender and country on the three growth parameters

**Parameters**	**Estimate**	**SE**	***t*** **-value**	***P*** **-value**	**95% CI**	
Asymptote β1	171.78	0.2356	728.99	0.0001	171.32	172.24
Gender (reference category=Female)					
Male asymptote (slope)	17.8564	0.5189	34.41	0.0001	16.8393	18.8736
Country (reference category=Ethiopia)					
India asymptote (slope)	-1.4132	0.1898	-7.45	0.0001	-1.7853	-1.0412
Peru asymptote (slope)	-0.4786	0.0829	-5.78	0.0001	-0.641	-0.3162
Vietnam asymptote (slope)	2.5338	0.1975	12.83	0.0001	2.1467	2.9208
Scale parameter β2	1.6662	0.0048	350.21	0.0001	1.6568	1.6755
Male scale parameter (slope)	0.1355	0.0060	22.57	0.0001	0.1237	0.1473
India scale parameter (slope)	-0.043	0.0057	-7.58	0.0001	-0.0541	-0.0319
Peru scale parameter (slope)	0.0017	0.0056	0.3	0.7631	-0.0092	0.0125
Vietnam scale parameter (slope)	0.0240	0.0055	4.4	0.0001	0.0133	0.0348
Rate of growth β3	0.1768	0.0007	248.17	0.0001	0.1754	0.1782
Male rate of growth (slope)	-0.0307	0.0009	-33.48	0.0001	-0.0326	-0.0289
India rate of growth (slope)	-0.0020	0.0007	-3.08	0.0021	-0.0033	-0.0007
Peru rate of growth (slope)	0.0036	0.0006	5.73	0.0001	0.0024	0.0048
Vietnam rate of growth (slope)	0.0009	0.0007	1.38	0.1681	-0.0004	0.0023

**Table 4:** Correlation of parameter estimates

	β1	β2	β3
β1	1.000		
β2	0.373	1.000	
β3	-0.668	-0.03	1.000

To understand the differences in growth characteristics between four low- and middle-income nations, as well as males and females, countries and gender, were treated as fixed effects on each parameter of the growth curve. Additionally, the asymptotic height and rates of growth varied across individuals and are considered random effects in analyzing the growth trajectories. The estimated parameters determined by the Logistic growth curve for females are β1=171.78, β2=1.666, and β3=0.177. The asymptotic height indicates, on average, that the maximum adult height for females is 171.78. The value related to the scale parameter β2 represents the ratio of height gained, and the value related to the parameter β3 is the rate of change that shows how fast females approach the asymptotic height. Males showed a substantial positive slope for asymptote (β1=17.856,P<0.001) and scale parameters (β2=0.136,P<0.001); however, they revealed a significant negative slope for the rate of growth (β3=-0.031,P<0.001). This indicates that males showed larger asymptotic height and scale parameters than females; nonetheless, females attain adult height earlier than males. This could be due to structural and anatomical differences between males and females^24^.

 The growth parameters are significantly affected by nation levels and indicate that the growth trajectories of children differ by country. Accordingly, the estimated asymptote slopes reported for India, Peru, and Vietnam are -1.413, -0.479, and 2.534, respectively. These are the estimated asymptotes difference between the asymptotes of the reference group (Ethiopia) and the corresponding countries. The negative asymptote slopes for children in India and Peru suggest that children in these two countries had lower asymptotic heights than children in Ethiopia. The positive asymptote slope for Vietnam indicates that children in Vietnam had greater asymptotic height, compared to those in Ethiopia. In terms of the growth rate, Peruvian and Vietnamese children had positive slopes, whereas Indian children had negative slopes. The mean rate of change for Ethiopian children was higher than that of Indian children and lower than that of Peruvian children. Children in Ethiopia approached adult height earlier than children in India; however, it was later than children in Peru. On the other hand, for children in Vietnam, the slope was not statistically significant. This indicates that children in Ethiopia and Vietnam have no significant differences regarding approaching adult height.

The correlations between parameter estimates were negative, except for those between the asymptotic height and scale parameter that were positive. The negative correlation between asymptotic height and rate of growth (𝑟𝛽1𝛽3 = -0.668) indicates that children with lower asymptotic height grow faster than those with higher asymptotic height. Similarly, the negative correlation between scale parameters and rate of growth (𝑟𝛽2𝛽3 = -0.0302) suggests that children with lower scale parameters grow faster than those with higher scale parameters. The biological correlation between asymptote and rate of growth is most important in growth curves^24^.

## Discussion

This study investigated various growth curve approaches to analyze children’s growth trajectories. Our concern was to fit the three-parameter growth curve that adequately describes the growth trajectories. Three-parameter growth curves are flexible curves for comparing and summarizing physical growth. However, growth curves with many parameters may lead to model fitting challenges^25^. The nonlinear growth curve attempts to estimate between-individual variations in within-individual change. It is more sophisticated for longitudinal data that follow nonlinear time trends^26^. Therefore, the families of three-parameter nonlinear growth curves in the context of the mixed-effects model were fitted to analyze the growth trajectories. The profile plots presented in Figures 1 and 2 confirmed the trends of height growth that were curvilinear. Accordingly, the nonlinear function is a reasonable function to model the growth trajectories for the current data.

In this study, the three-parameter growth curves were introduced to analyze child’s growth trajectories. Comparisons of models’ goodness of fit and selection procedures were carried out according to the goodness of fit indicators, residual distribution of the models, and biologically meaningful growth parameters. In addition to comparing the growth curves that best fit the height growth using the goodness of fit indicators, considering the biological expectation of the growth parameters is also helpful ^23^. The plots of residuals against age for both the Gompertz and Logistic curves showed similar trends with no strong association over time. This indicates that both curves can achieve the growth trajectories well. However, the Gompertz curve overestimated the asymptotic height. The mean adult height provided by the Logistic curve is the biological expected mean adult height. Therefore, for the current data, the Logistic growth curve was preferred to analyze the growth trajectories. Lampl^27^ noted that the Logistic and Gompertz curves were the most common mathematical functions used to model human growth as a function of age.

The Von Bertalanffy curve is the worst model for the current data. Ahmadi et al.^22^ compared three growth curves (i.e., the Jenss, the Reed, and the Gompertz curve) with the height of children from birth to age of six and reported that the Gompertz curve did not fit well for both males and females. However, they did not include the other three-parameter growth curves rather the Gompertz curve. To capture between- and within-individual growth patterns, the fixed and random effects were considered in the Logistic growth curve.

 Children varied across their asymptotic height and rate of growth. The effects of gender and country on the three parameters of the Logistic curve were examined. The model provided that the inclusion of covariates in the growth modeling process considerably reduced the values of fit statistics. Both covariates had significant effects on all growth parameters.

Males had significantly higher asymptotic height and scale parameters but had a lower rate of growth than females. Females reached the asymptotic height faster than males. India and Peru have significantly negative slopes for asymptotes, while Vietnam has a positive slope. The mean asymptotic height of children in Ethiopia is higher than that of children in India and Peru but lower than the mean asymptotic height of children in Vietnam. Children in Ethiopia reached adult height faster than those in India but lower than children in Peru. The differences in the growth trajectories among children in these countries could be due to socioeconomic differences^28^. These differences in height growth existed from birth and expanded during infancy and early childhood^29^. Children from better socioeconomic backgrounds were taller than those from lower socioeconomic backgrounds^30^. On the other hand, the growth variations may be due to precocious puberty. The primary issue with precocious puberty is the height since precocious puberty causes rapid development, accelerated bone maturation, and eventually decreased stature. Precocious puberty is rare in boys ^31^, and causes children to grow fast initially and be taller than their peers. However, since their bones mature at a faster rate than normal, they typically cease growing sooner than expected. As a result, they may grow up to be shorter than usual.

 There are certain limitations to our study which mainly emanated from the limitedness of factors and covariates captured in this study. However, it can be acknowledged that certain other variables, such as a child’s nutritional condition, birth weight, and health-care utilization, to name a few, would have made the study more comprehensive. In future research, the model employed in this study may simply be extended to numerous time-invariant and time-varying variables.

## Conclusion

 In conclusion, this study examined the performances of different nonlinear growth curves for the growth trajectories of children in Ethiopia, India, Peru, and Vietnam. The nonlinear Logistic curve was a better fitting curve for modeling the growth trajectories. Gender and country have significant effects on the three-parameter of the curve. Further enhancements may be attained with the inclusion of other potential covariates.

## Acknowledgments

 The authors thank the Young Lives study for giving access to the data files.

## Conflict of Interest

 The authors declare that there is no conflict of interest.

## Funding

 None.

 Highlights

- The Logistic curve was found to be the best growth curve to describe the growth trajectories.
- Gender and country differences had significant effects on the child’s growth.
- Children in Ethiopia reached adult height faster than those in India but lower than children in Peru.
- Females reached asymptotic height earlier than males.
